# Fracture Risk in Chronic Kidney Disease: Addressing an Overlooked Complication

**DOI:** 10.3390/metabo15070460

**Published:** 2025-07-07

**Authors:** Guido Gembillo, Concetto Sessa, Walter Morale, Luca Zanoli, Antonino Catalano, Salvatore Silipigni, Luca Soraci, Andrea Corsonello, Maria Princiotto, Carlo Lomonte, Domenico Santoro

**Affiliations:** 1 Unit of Nephrology and Dialysis, Department of Clinical and Experimental Medicine, University of Messina, 98125 Messina, Italy; dsantoro@unime.it; 2 Nephrology and Dialysis Department, “Maggiore” Hospital, Modica, 97100 Ragusa, Italy; walter.morale@asp.rg.it; 3 Department of Clinical and Experimental Medicine, University of Catania, 95100 Catania, Italy; luca.zanoli@unict.it; 4 Unit and School of Geriatrics, Department of Clinical and Experimental Medicine, University Hospital of Messina, Via C. Valeria, 98125 Messina, Italy; catalano.antonino@unime.it; 5 Department of Biomedical Sciences and Morphologic and Functional Imaging, Policlinico “G. Martino”, University of Messina, Via Consolare Valeria 1, 98100 Messina, Italy; salvatore.silipigni@polime.it; 6 Unit of Geriatric Medicine, Italian National Research Center on Aging (IRCCS INRCA), 87100 Cosenza, Italy; l.soraci@inrca.it (L.S.); a.corsonello@inrca.it (A.C.); 7 Department of Pharmacy, Health and Nutritional Science, University of Calabria, 87036 Rende, Italy; 8 Laboratory of Pharmacoepidemiology and Biostatistics, Italian National Research Center on Aging (IRCCS INRCA), 87100 Cosenza, Italy; m.princiotto@inrca.it; 9 U.O.C. di Nefrologia e Dialisi, Ospedale Generale “F. Miulli”, 70021 Acquaviva delle Fonti, Italy; carlolomonte@libero.it

**Keywords:** fracture risk, dialysis, CKD-MBD, renal osteodystrophy, bone health

## Abstract

Fracture risk is a serious yet underrecognized complication among patients with chronic kidney disease (CKD), especially in those with stages G3-G5D. The overlap between CKD-Mineral and Bone Disorder (CKD-MBD) and osteoporosis leads to complex bone changes that increase the likelihood of fragility fractures. Studies show that 18% to 32% of CKD patients also have osteoporosis, and these individuals are more than 2.5 times as likely to suffer from fractures compared to those without CKD. In the advanced stages of the disease, fracture risk is up to four times higher than in the general population, with the femur, forearm, and humerus being the most commonly affected sites. Hip fractures are of particular concern as they are linked to longer hospital stays and higher rates of morbidity and mortality. Furthermore, dialysis patients who experience hip fractures have a mortality rate 2.4 times higher than those in the general population with similar fractures. This increased risk underscores the need for proactive bone health maintenance in CKD patients to prevent fractures and related complications. This review explores the underlying pathophysiological mechanisms, diagnostic challenges, and treatment options related to bone fragility in CKD. Diagnostic tools, such as bone mineral density (BMD) assessments, the trabecular bone score (TBS), and biochemical markers, remain underused, especially in advanced CKD stages. Recent treatment strategies emphasize a multidisciplinary, stage-specific approach, incorporating calcium and vitamin D supplements, anti-resorptive agents like denosumab, and anabolic therapies such as teriparatide and romosozumab. Effective management needs to be tailored to the patient’s bone turnover status and stage of CKD. Despite progress in understanding bone fragility in CKD, significant gaps remain in both diagnosis and treatment. Personalized care, guided by updated KDIGO recommendations and based on an interdisciplinary approach, is essential to reduce fracture risk and improve outcomes in this vulnerable population. Further research is needed to validate risk assessment tools and refine therapeutic protocols.

## 1. Introduction

Fractures are a well-known and dramatic consequence of bone fragility in chronic kidney disease (CKD), contributing significantly to morbidity and mortality. Beyond their clinical impact, fractures also substantially impair quality of life and increase healthcare costs for patients with CKD [1]. Strategies to identify patients at risk through an appropriate evidence-based management can prevent or mitigate these serious skeletal complications. Although mineral and bone disorder related to chronic kidney disease (CKD-MBD) is one of CKD major complications, osteoporosis and related fractures issues have long been neglected in the nephrology community and still remain an unmet clinical need. Yet, CKD is an independent risk factor for osteoporosis, and the latter has a high prevalence in CKD [2,3]; furthermore, the incidence of fractures is significantly higher in patients with CKD stages G4-G5D, especially among older adults aged over 65 years and other frail subgroups [4,5].

Importantly, major osteoporotic fractures (the hip, spine, humerus, and forearm) can affect CKD patients during the transition period to dialysis, spanning from one year before to one year after dialysis initiation [6]. Notably, these patients may present with normal serum vitamin D, parathyroid hormone (PTH), calcium and phosphate [2], making clinical vigilance essential.

In 2017, KDIGO Guidelines (CKD-MBD Update Work Group), for the first time, suggested the assessment of bone mineral density (BMD) in CKD G3-5D patients with risk factors for fractures, highlighting the clinical relevance of osteoporosis in this population [7]. Subsequently, the term “CKD-associated osteoporosis” was frequently used, and more recently, the new term “CKD-MBD/osteoporosis” has been proposed, placing osteoporosis into the official broader CKD-MBD spectrum [8]. However, despite increased awareness, we need a strong call to action. Nephrologists, together with other bone specialists, should develop and lead a multidisciplinary osteoporosis team to counteract the enormous impact that bone fragility can have on patients’ quality of life and healthcare costs [1].

The aim of this review is to provide a complete and up-to-date overview of the pathophysiology, diagnosis and management of CKD patients easily exposed to the risk of fracture, and this manuscript is structured as a narrative review based on a comprehensive synthesis of the relevant peer-reviewed literature.

## 2. Osteoporosis in Chronic Kidney Disease: A Rising Comorbidity in an Aging Population

The global prevalence of CKD is steadily increasing, accordingly with population aging, posing a growing public health challenge. Osteoporosis is a common comorbidity in patients with CKD, particularly in advanced stages. Indeed, it is twice as prevalent among those with an eGFR less than 60 mL/min/1.73 m^2^ compared to individuals with preserved kidney function and affects up to 30% of patients in stages G3-G5 [2]. This increased risk is largely attributable to impaired calcium and phosphate metabolism, secondary hyperparathyroidism, and vitamin D deficiency [9]. By 2030, nearly 2 million people worldwide are projected to be receiving chronic dialysis, a population with a 3- to 8-fold increased risk of bone fractures compared to the general population [10]. The incidence of CKD in the older population is rising, particularly in industrialized countries. Although cardiovascular and infectious diseases remain the leading causes of death among dialysis patients, systemic complications such as osteoporosis have recently gained greater recognition [9]. Systematic reviews have highlighted the increasing global burden of fractures, with hip fracture rates in women ranging from 100 to 600 per 100,000 inhabitants and vertebral fracture rates from 100 to 1400 per 100,000, varying by region. The highest hip fracture rates are found in Scandinavian countries (26%) and the lowest in Eastern Europe (18%). Vertebral fracture rates are most prevalent in South Korea and the United States and least common in the United Kingdom. In the United States, these issues are more common in the white population than in the black population [11,12].

Nephrologists frequently encounter bone health concerns in their patients, particularly osteoporosis in older CKD patients, who often have additional risk factors such as smoking, diabetes mellitus, and heart failure. As renal function declines, CKD-specific alterations in bone metabolism emerge, primarily driven by secondary hyperparathyroidism [13]. In the early stages of CKD, the decreased synthesis of 1,25-dihydroxyvitamin D and phosphate clearance lead to the overstimulation of the parathyroid glands and vascular calcifications, which are linked to increased cardiovascular mortality. These biochemical changes constitute the CKD–mineral bone disorder (CKD-MBD), a condition that can progress to renal osteodystrophy [13]. The coexistence of osteoporosis and CKD presents a significant clinical challenge, particularly with the rising fracture incidence in aging populations. Despite the elevated fracture risk, patients with CKD stages G3-G5D are often underdiagnosed and undertreated, mainly because they are excluded from major clinical trials. Bone fragility in CKD is multifactorial and influenced by premature kidney aging, chronic wasting, and disturbances in vitamin D/mineral metabolism. Embedding osteoporosis management within CKD-MBD guidelines is essential, though there remains an urgent need for dedicated clinical trials to inform evidence-based fracture prevention strategies in this vulnerable population [14].

The primary aim of this review is to provide an updated and comprehensive overview of the current state of the art of the therapeutic approaches to bone fragility and fracture risk in patients with CKD. Given the high prevalence of bone mineral density loss and the increased fracture risk in this population, a thorough understanding of diagnostic strategies and treatment options is critical for improving patient outcomes.

## 3. Overview of Osteoporosis: Definition and Core Concepts

Definitions: Osteoporosis is characterized by reduced bone density and the deterioration of skeletal microarchitecture, leading to decreased bone strength and increased fracture risk, particularly in areas like the vertebral column, hip, and wrist. The World Health Organization defines osteoporosis as a bone mineral density (BMD) more than 2.5 standard deviations (SD) below the young adult mean, while most fractures occur in individuals with osteopenia (T-scores between −1.0 and −2.5 SD), who represent a larger population [15].

The concept of CKD-MBD was introduced in 2006 by the Kidney Disease: Improving Global Outcomes (KDIGO) [16] project to summarize the clinical syndrome characterized by (a) phosphate retention; (b) reduced serum vitamin D3 concentration due to decreased activation to 1,25-dihydroxyvitamin D by 1-α-hydroxylase, which declines for filtrates <60 mL/min/1.73 m^2^; (c) calcium anomalies (hypocalcemia likely due to hyperphosphatemia, PTH resistance, and reduced active vitamin D3 concentration); (d) increased serum FGF23 in response to reduced Klotho receptors; (e) bone resistance to PTH (secondary and tertiary hyperparathyroidism); and (f) extraskeletal calcifications. These bone metabolism anomalies worsen the quality of life and increase cardiovascular mortality. KDIGO aims to develop a clear, internationally valid classification system based on laboratory markers and imaging for clinical patient evaluation [16].

The traditional term “Renal Osteodystrophy” (ROD) represents the phenotypic expression of CKD-MBD metabolic alterations on bone that affects virtually all adults and children with chronic kidney disease [2,17]. Within bone pathology (turnover, mineralization, growth, and bone strength anomalies), this term should be reserved for identifying specific histomorphometric patterns detected by biopsy [18].

KDIGO has identified three osteodystrophy markers represented by the TMV system (turnover, mineralization, and volume) [6]: (a) osteitis fibrosa cystica: high bone turnover and hyperparathyroidism; (b) adynamic bone disease: low turnover due to PTH suppression; and (c) osteomalacia: low bone turnover and slow bone mineralization (less frequent due to the reduced prescription of aluminum-based phosphorus binders and improved dialysis concentrates) [13]. The literature reports a relationship between fragility fractures, osteoporosis, and CKD-MBD, leading to increased social costs, morbidity, and mortality in CKD patients [19].

## 4. Pathophysiological Mechanisms Underlying Fragility Fractures in CKD Patients

The pathophysiology of fragility fractures in CKD is multifactorial [20], involving imbalances in trabecular bone structure, disorganized turnover, and altered mineralization. The main pathophysiological mechanisms underlying bone fractures in CKD are summarized in Table 1.

**Figure 1:**
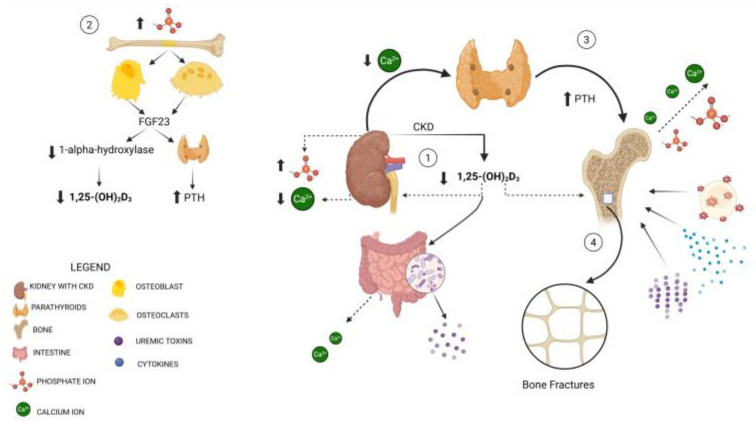
Pathophysiological mechanisms linking chronic kidney disease (CKD) to bone fragility. The figure illustrates the complex interactions between kidney dysfunction and bone fragility in the context of chronic kidney disease (CKD). The pathway begins with reduced kidney function, which leads to impaired phosphate excretion and decreased active vitamin D (1,25-dihydroxyvitamin D) production with lower intestinal calcium absorption (1). Osteoblasts and osteoclasts increase FGF23 secretion to maintain a neutral phosphate balance, but this results in decreased 1-alpha-hydroxylase synthesis and the suppression of 1,25-dihydroxyvitamin D production with the development of secondary hyperparathyroidism. This disruption causes an imbalance in calcium and phosphate homeostasis, promoting secondary hyperparathyroidism. Elevated parathyroid hormone (PTH) levels stimulate bone resorption, further exacerbating bone loss. Additionally, CKD-induced inflammation, oxidative stress, and the accumulation of uremic toxins (metabolized by the intestinal flora) contribute to bone mineralization defects and alterations in the bone microarchitecture. The combination of these factors leads to a significant increase in bone fragility, predisposing CKD patients to fractures. This complex interplay highlights the importance of early intervention in managing bone health in CKD. Legend: CKD = chronic kidney disease; PTH = parathyroid hormone; PO_4_^3−^ = phosphate ions; FGF23 = fibroblast growth factor 23; Ca^2+^ = calcium ions.

Bone health is closely linked to kidney health, with bone strength determined by bone mineral density (BMD) and bone quality (BQ). BMD measures the mineral content in bone, while BQ includes characteristics like microarchitecture, the turnover rate, and the presence of micro-damage. Calcium and phosphate homeostasis is primarily regulated by epithelial calcium and phosphate cotransport in the kidneys and intestines, under the influence of hormones such as 1,25-dihydroxyvitamin D (1,25(OH)2D), fibroblast growth factor 23 (FGF23), and PTH. In patients with CKD, declining kidney function disrupts mineral homeostasis, finally causing bone loss and fragility fractures [21], while cardiovascular disease, exacerbated by vascular calcification [22], remains a significant complication in CKD [5,23]. Uremic toxins, such as indoxyl sulfate and AGEs, contribute to impaired BQ and skeletal resistance to PTH, leading to increased bone fragility. Microdamage accumulation due to low bone turnover further elevates fracture risk [24], as illustrated in Figure 1. Vitamin K deficiency is common in patients with CKD and contributes to both cardiovascular disease and bone fragility. Vitamin K plays a crucial role as a post-translational cofactor for the γ-carboxylation of vitamin K-dependent proteins including osteocalcin, which are essential for inhibiting vascular calcification and supporting bone health. Poor vitamin K status is linked to the progression of chronic kidney disease and its associated complications, indicating a potential benefit of vitamin K supplementation, though further studies are needed to determine the appropriate dosage [25]. In CKD, the loss of muscle mass, as well as the loss of muscle strength and low physical performance, are also commonly observed and may lead to sarcopenia as a result of the accelerated protein catabolism; this condition enhances the risk of falls and fractures, particularly in older patients [26].

## 5. Diagnostic Management

Understanding the mechanisms of bone loss is crucial for preventing fractures, especially for the diagnosis of fractures in patients with CKD, which requires a multifaceted approach, integrating clinical evaluation with biochemical, imaging, and histological assessments (Table 2).

### 5.1. Bone Biomarkers

Bone fragility in CKD is a multifactorial condition resulting from altered mineral metabolism, the reduced activation of vitamin D, phosphate retention, hypocalcemia, and secondary hyperparathyroidism. These factors disrupt the normal balance of bone formation and resorption, leading to impaired bone quality and increased fracture risk, particularly in advanced stages of CKD and among dialysis patients. Managing bone pathology is more complex in CKD patients compared to the general population of the same age because of abnormalities in calcium, phosphorus, PTH, and vitamin D, with epiphenomena including vascular and soft tissue calcifications and alterations in bone physiology, turnover, and mineralization [13]. Biochemical markers are pivotal in distinguishing between various forms of bone disease. Serum calcium, phosphate and PTH levels are routinely monitored to assess abnormalities in mineral metabolism. Elevated PTH levels, especially in advanced CKD, often point towards secondary hyperparathyroidism, which can predispose patients to high-turnover bone disease, such as osteitis fibrosa. Conversely, low PTH levels may suggest adynamic bone disease, a condition of low bone turnover. Bone-specific alkaline phosphatase (BSAP), a marker of bone formation, is particularly useful in differentiating high from low bone turnover. Fibroblast growth factor 23 (FGF23), a protein produced by mature osteoblasts [27], plays a role in mineral homeostasis by binding to its receptor complex, FGFR/Klotho, primarily located in the kidneys. It is considered one of the newest biomarkers of bone dysfunction, but its serum levels can be influenced by various factors, such as inflammatory processes, iron status, hypoxia, heart failure, or erythropoietin concentration, all of which can negatively impact mortality. Additionally, active vitamin D levels are typically low in CKD due to the impaired synthesis of 1,25-dihydroxyvitamin D, which further contributes to altered bone metabolism and increased fracture risk [7,28,29]. The interconnections between all bone biomarkers involved in mechanisms underlying bone fracture risk in CKD are reported in Figure 1.

### 5.2. Fracture Risk Tools in CKD

The Fracture Risk Assessment Tool (FRAX) is an algorithm, introduced in 2008, that estimates the 10-year probability of major osteoporotic fractures (the spine, forearm, hip, or shoulder) based on a scoring system that combines independent clinical risk factors, such as age, sex, body mass index, previous fragility fracture, a parental history of hip fracture, the use of glucocorticoids, rheumatoid arthritis, secondary osteoporosis, current smoking, and an alcohol intake of three or more units per day [30]. In an Italian study conducted on postmenopausal women attending an osteoporosis center, the systematic use of FRAX and clinical parameters allowed the discovery of a high prevalence of unrecognized fracture risk, underlining the importance of targeted screening strategies in the outpatient setting as well [31]. The use of T-scores in relation to fractures in patients receiving HD is recognized and well-established. Although the FRAX prognostic threshold for the identification of an increased risk of major bone fractures in hemodialysis patients is >5% [32], and it should be used as an intervention threshold for pharmacological antifracture treatment in HD patients, there is a paucity of data regarding the additional contributions of FRAX in assessing frailty status and falls and their its relationship with fracture [33]. An important update to the FRAX score is FRAXplus, introduced in 2024. FRAXplus is an improved version of the standard FRAX tool that allows for a more refined risk assessment by incorporating additional clinical information and adjustments into the FRAX model. While the standard FRAX tool primarily uses 11 standard clinical risk factors and bone mineral density (BMD) to predict fracture risk, FRAXplus can also predict fracture probability based on factors such as the recency of osteoporotic fractures, the duration of type 2 diabetes, glucocorticoid use, fall history, trabecular bone score (TBS) information, concurrent lumbar spine BMD and hip axis length (HAL) information [34]. Italian bone specialists have developed and updated the Derived Fracture Risk Assessment (DeFRA) tool. The DeFRA algorithm provides an assessment of the different fracture risk factors that align with those considered by Italian health authorities such as the Istituto Superiore di Sanità (ISS) and the Agenzia Italiana del Farmaco (AIFA). DeFRA has been endorsed by the Italian Society of Osteoporosis, Mineral Metabolism and Skeletal Diseases (SIOMMMS), the Italian Society of Rheumatology (SIR) and the Italian Bone Interdisciplinary Specialists Group (GIBIS). The latest version of the DeFRA algorithm includes a number of comorbidities; amongst them are CKD and other conditions that were not previously considered such as neurological disorders like dementia and Parkinson’s disease. This enhanced tool moves beyond simple dichotomic answers and provides detailed responses regarding the frequency of previous fractures and the use of medications that increase fracture risk, such as adjuvant hormone therapy, in addition to corticosteroids [35]. Fusaro et al. introduced Kidney Derived Fracture Risk Assessment (K-DeFRA) in 2017. K-DeFRA considers the different stages of CKD and distinguishes between various dialysis methods. It has been observed that fracture risk is higher in patients undergoing conventional hemodialysis (HD) compared to those on nocturnal hemodialysis (HDN) and peritoneal dialysis (PD), offering a more tailored approach to fracture risk assessment in CKD patients [5].

The association between bone quality, muscle strength and fracture risk has recently been confirmed in the dialysis setting and through the use of tools such as the trabecular bone score and quantitative phalangeal ultrasonography (QUS), which have proven to be useful predictors of skeletal fragility in hemodialysis patients [36].

While FRAX, DeFRA, and K-DeFRA represent valuable tools in the general population, their application in patients with advanced CKD remains problematic. These models do not incorporate key CKD-specific factors such as bone turnover status, vascular calcification, or dialysis vintage, and their predictive performance in this population is poorly validated. As a result, clinicians may either over- or underestimate fracture risk. There is an urgent need for tailored risk assessment models that reflect the complex interplay between mineral metabolism, comorbidity burden, and functional status in CKD. Future research should prioritize the development and validation of such tools to improve fracture prevention strategies in this high-risk population.

In patients with advanced CKD, bone diagnostic tools present unique challenges. While DXA remains the most accessible method for assessing BMD, its utility in CKD G4–G5D is limited. Vascular calcifications, degenerative changes, and fluid overload can artificially elevate BMD readings, especially at the lumbar spine. Similarly, TBS, though promising, may be affected by body composition changes and lacks validation in dialysis patients. In clinical practice, a low BMD or degraded TBS should raise suspicion for increased fracture risk, but normal values do not exclude bone fragility in this population. Where available, HR-pQCT offers superior resolution and can distinguish cortical and trabecular defects, although its accessibility is limited. In settings lacking advanced imaging, the integration of biochemical markers of bone turnover (e.g., PTH, bone-specific ALP, PINP, CTX) alongside clinical risk factors and fracture history remains crucial. Ultimately, diagnosis in CKD must balance technological limitations with a contextual, multimodal approach [36].

## 6. Radiologic Imaging

The lack of the specificity of humoral biomarkers encouraged the development of dedicated non-invasive techniques to assess changes in bone quantity, microarchitecture and mineral composition. In this context, radiologic imaging gained a decisive role thanks to its repeatability and relative non-invasiveness.

### 6.1. Conventional Radiography

Despite its worldwide availability, conventional radiography is insufficient for the monitoring of BMD due to the intrinsic limitation of qualitative evaluation and the impossibility to score bone mineralization and its modifications. However, conventional radiography represents the first choice for the identification of fractures in a symptomatic patient in cases where pain, trauma or a suspected fracture is present [37]. In the case of patients with augmented bone fragility like CKD patients, a conventional radiologic study can show signs of bone remodeling. These phenomena are very dependent on the anatomic area: sub-periosteal resorption is commonly found at distal phalanges, while sub-tendinous resorption is more common at trochanter and ischial tuberosity [38]. Other signs of systemic bone remodeling in cases of CKD are the “salt and pepper” appearance of skull bones or radiolucent areas of focal bone resorption with endostal scalloping, normally referred to as brown tumors, which tend to be localized in long bones, ribs and pelvic bones [37].

An early x-ray assessment of symptomatic areas can also reveal areas of osteomalacia, also known as stress fractures, visible as linear radiolucent bands perpendicular to the cortex, which represent a radiologic manifestation of incomplete fractures. Extraosseous manifestations of CKD-BMD are also visible upon using a conventional X-ray, appearing as extra skeletal calcifications (chondrocalcinosis, tumoral calcinosis and vascular calcifications) [37,39].

### 6.2. DXA

Bone mineral density can be evaluated using dual-energy X-ray absorptiometry (DXA) or high-resolution peripheral quantitative computed tomography (HR-pQCT), which is a valuable tool for assessing bone geometry and microarchitecture in CKD patients, providing insights into the structural changes that predispose these patients to fractures [4]. Nowadays, DXA, implemented into clinical use in 1987, represents the best tool for BMD measurement. Using two X-ray beams at different kilovoltage peak potential values (conventionally 30 to 50 and 70 keV), DXA allows an areal BMD measurement of the lumbar spine (L1–L4 vertebral bodies), femur (the neck and trochanteric region), and distal radius, with the subtraction of soft tissues. After an operator supervises automatic segmentation, DXA provides areal density values (g/cm^2^), Z scores (standard deviations, with comparisons made among age- and gender-matched reference populations) used for premenopausal women, men up to 50 yr and children, and T scores (standard deviations, with comparisons made among the young adult reference population) [40,41]. The advantages of this technique are its low radiation dose, worldwide availability and low costs. Unfortunately, DXA has known limitations, especially when performed on CKD patients: in fact, as a bidimensional imaging method, it is not able to discriminate patient-specific characteristics like spinal degenerative changes and the presence of calcified vascular plaques in major abdominal vessels (aorta and iliac arteries) that could overlap in the analyzed area, often leading to an overestimation of BMD; such limits are not present with volumetric techniques such as CT. These pitfalls unavoidably increase the risk of underestimating depletive bone remodeling and related fracture risks.

Bone quality can also be assessed through the trabecular bone score (TBS), a textural index that evaluates pixel gray-level variations in the lumbar spine DXA image and provides an indirect indicator of bone microarchitecture, high-resolution QTC bone imaging and bone biopsy. Although the 2009 KDIGO guidelines recommended against routinely measuring BMD with DXA in patients with CKD stages G3a-5D due to a lack of evidence on its predictive value for fracture risk [42], revised recommendations in 2017 marked a shift in opinion. These updated guidelines suggest that assessing BMD in CKD stages G3a-G5D can indeed predict longitudinal bone loss that occurs at the proximal radius [43] and fracture risk, particularly when the results influence treatment decisions [44]. BMD measurement via DXA has proven valuable in predicting fracture risk across the full spectrum of CKD stages G3a to 5D. For the first time, guidelines have recommended BMD assessment in CKD G3-5D patients who exhibit CKD-MBD and/or fracture risk factors, provided that the results have the potential to influence therapeutic decisions. This approach underscores the critical role of BMD evaluation in managing osteoporosis in CKD patients [45,46].

### 6.3. Computed Tomography (CT)

By providing volumetric BMD measurements, CT has overcome some of the limitations of bidimensional planar imaging. Available techniques for BMD measurement are quantitative computed tomography (QCT) and high-resolution peripheral QCT (HR-pQCT). Volumetric reconstructions allow the differentiation between trabecular and cortical bone and also avoid interference from extraskeletal calcifications and degenerative changes to the lumbar spine or other confounding conditions like scoliosis. Central QCT involves an areal scan of the lumbar spine or hip, and (through calibration with dedicated phantoms) allows a quantitative and morphological evaluation of trabecular-rich regions, areas preferentially affected in CKD-related bone loss. This makes it less susceptible to artifacts, but its significantly higher radiation dose compared to DXA limits its use to specific cases [37].

HR-pQCT requires dedicated scanners for BMD volumetric measurement in peripheral areas such as the distal radius and tibia. It carries all the advantages of volumetric bone assessment with a significantly lower administered radiation dose. It allows an assessment of trabecular and cortical bone architecture with a quantitative assessment of trabecular thickness, number, separation, and cortical porosity—parameters not captured by standard QCT. While HR-pQCT has demonstrated strong correlations with fracture status in both postmenopausal women and elderly men, its role in CKD remains to be explored. Studies have suggested that cortical deterioration and increased porosity, especially in type 2 diabetic patients—a group commonly overlapping with CKD—may be early indicators of skeletal fragility despite preserved BMD. Nevertheless, HR-pQCT is currently confined to research settings due to its limited availability, high cost, and restriction to peripheral sites [40].

### 6.4. Magnetic Resonance Imaging (MRI)

MRI use has also been investigated for bone trabecular architecture evaluation in the past, but its complex scanning protocols and reduced availability have led to the preferred use of other techniques. In addition, the very short relaxation time of the mineralized matrix has previously limited a direct evaluation of bone structure. On the other hand, through specific sequences such as water–fat chemical shift MRI, quantitative susceptibility mapping and MRI spectroscopy, MRI enables the radiation-free quantification of bone marrow fat fraction fat saturation and water content [41].

The recent development of ultrashort echo time (UTE) or zero echo time sequences have allowed us to acquire rapidly decaying MRI signal from the bone matrix, enabling a direct MRI evaluation with the quantification of bone water content. Comparative studies with DXA and HR-pQCT demonstrated increased water content in patients with osteoporosis due to the decreased mineralization of the bone matrix with a negative correlation against BMD obtained with DXA and HR-pQCT [47,48]. These parameters may be correlated to impaired bone strength due to the increased porosity of cortical bone in sensitive anatomic areas such as the proximal femur in vivo. Despite these capabilities, the clinical use of MRI in bone fragility assessment remains limited, mainly due to its long acquisition times, reduced availability and low spatial resolution compared to CT-based methods and the need for specialized sequences.

### 6.5. Quantitative Ultrasound (QUS)

QUS represents another non-ionizing and low-cost technique for the assessment of fracture risk. It bases its principle on the quantitative analysis of the propagation of ultrasound waves through cortical bone. The measurement of speed of sound waves conduction through bone and the broadband attenuation of wave quantification allows the estimation of bone density modification. Literature studies have demonstrated a correlation between osteoporotic fracture risk and decreased ultrasound cortical speed in a normal population and in dialysis patients with secondary hyperparathyroidism [49,50]. QUS is conventionally measured at the calcanean, tibial or phalangeal surface, but measurements are solidly validated only at heels; however, the variability in measurement with the different measurement sites and different existing scanner types, the lack of validation and the limited diffusion of dedicated scanners still restrain QUS use at the research level [41].

### 6.6. Nuclear Medicine

Molecular imaging modalities are able to map the accumulation or radionuclide-marked bone metabolites with high sensitivity, allowing the early detection of bone metabolic uptake changes due to impaired or increased osteobastic activity in patients with osteoporosis [34]. Different tracers have been studied in the past few decades, and nowadays, the most promising application for osteoporosis assessment is represented by 18F-sodium fluoride (NaF) PET/CT [41,51]. NaF uptake reflects osteoblastic activity and is closely associated with regions of high bone remodeling. In a glucocorticoid-induced postmenopausal osteoporosis cohort study, NaF-PET showed high sensitivity in capturing NaF uptake modification after 3 months of treatment with alendronate administration, anticipating any BMD modification [52]. However, the interpretation of tracer kinetics in CKD patients may be complicated by an altered phosphate metabolism and prospective studies are necessary to validate the role of NaF in extensive clinical use. Moreover, despite the promising results, logistical challenges, high cost, and limited access to PET tracers (cyclotron preparation) currently restrict their use to research settings. Although bone biomarkers and imaging techniques are commonly used to assess bone health in CKD patients, they have inherent limitations. In cases where these methods provide inconclusive results, bone biopsy remains the gold standard for definitive diagnosis. A transiliac bone biopsy, combined with histomorphometric analysis, allows for the direct evaluation of bone turnover, mineralization, and volume, which is crucial for distinguishing between the various forms of ROD, such as osteomalacia, osteitis fibrosa, and adynamic bone disease. This approach is particularly valuable when considering anti-resorptive therapy, as it guides appropriate treatment strategies based on the specific bone pathology. Despite its diagnostic value, bone biopsy is rarely utilized in clinical practice and research. To address this gap, the European Renal Osteodystrophy initiative was launched in 2016 to promote the use of bone biopsies and foster research on the epidemiology, clinical implications, and potential reversibility of ROD. By encouraging the use of this diagnostic tool, the initiative aims to improve the understanding of ROD and ultimately enhance patient outcomes in CKD, advocating for more comprehensive and targeted approaches to diagnosis and treatment [53].

## 7. Therapeutic Strategies

The therapeutic management of osteoporosis CKD requires a tailored approach due to the complex interplay between bone and mineral metabolism, particularly in advanced stages of CKD. To facilitate a clearer understanding and guide clinical decision-making, we have stratified therapeutic strategies based on the patients’ eGFR. The therapeutic options for patients with an eGFR ≥ 30 mL/min/1.73 m^2^ and eGFR < 30 mL/min/1.73 m^2^ and those undergoing hemodialysis (G5D) differ significantly due to variations in bone turnover and mineral metabolism. A summary of the main therapeutic options in distinct CKD stages is reported in Table 3.

### 7.1. Therapies Available for All CKD Stages

Calcium supplementation is commonly employed in the prevention of osteoporotic fractures, including among patients with CKD. The most widely used formulations are calcium carbonate, which contains 40% elemental calcium, and calcium citrate, which contains approximately 21% but offers distinct clinical advantages [54,55].

Calcium carbonate is cost-effective but requires an acidic gastric environment for optimal absorption, making it less effective in elderly patients or those on proton pump inhibitors. In CKD, excessive intake has been associated with hypercalcemia and vascular calcifications [16].

Calcium citrate, in contrast, is absorbed independently of gastric pH and is generally better tolerated, and its citrate component can increase urinary citrate—a known inhibitor of calcium stone formation and potential contributor to bone protection. In patients with mild metabolic acidosis, which is frequent in advanced CKD, citrate may help buffer acid load and reduce bone resorption. Recent evidence suggests that urinary citrate may serve as a noninvasive biomarker of systemic acid–base status and a predictor of bone strength. In a study by Esche et al., low urinary citrate levels were associated with impaired bone strength in adolescents and higher fracture risk in adult females. The authors concluded that chronic low-grade metabolic acidosis, reflected by reduced citraturia, could be detrimental to bone health from childhood into adulthood and that dietary or metabolic acid load may play a key role in long-term skeletal integrity [56]. The KDIGO 2017 guidelines recommend limiting the use of calcium-based phosphate binders, including calcium carbonate, in patients with CKD G3a–G5D to avoid calcium overload complications. However, they do not directly address the choice of calcium salt for bone health. In this context, calcium citrate may represent a preferred option in selected cases, such as recurrent stone formers, those with metabolic acidosis, or patients intolerant to carbonate [7]. In light of these findings, calcium citrate could represent a preferential choice in patients with CKD, particularly in the presence of reduced citraturia, not only to prevent nephrolithiasis but also to contribute to the maintenance of bone health. Its alkalinizing effect, in fact, counteracts chronic mild metabolic acidosis and promotes an environment favorable to bone mineralization, reducing the risk of fragility fractures.

### 7.2. Patients with eGFR ≥ 30 mL/min/1.73 m^2^ (CKD Stages G1–G3)

In patients with an eGFR ≥ 30 mL/min/1.73 m^2^, therapeutic management mirrors that of the general population with added caution for renal complications. The first line of treatment focuses on lifestyle modifications, including diet, smoking cessation, caution with the use of a proton pump inhibitor in patients without a strong indication [57], adequate calcium and vitamin D intake [58], and regular exercise to prevent further bone loss [59]. Pharmacologic therapy can be initiated if necessary [60].

• Bisphosphonates: Oral bisphosphonates such as alendronate and risedronate are the primary agents for reducing fracture risk in CKD G1–G3 patients. Studies have confirmed the efficacy of bisphosphonates in improving BMD and preventing fractures in CKD patients with preserved renal function. However, the risk of renal complications mandates close monitoring [61].
• Denosumab: Denosumab, a monoclonal antibody that inhibits RANKL, offers a promising alternative to bisphosphonates. It is safe in patients with an eGFR ≥ 30 mL/min/1.73 m^2^, as it is not excreted by the kidneys. However, the risk of hypocalcemia necessitates calcium monitoring, especially in patients with CKD, who may already have altered calcium metabolism [62]. Recent data suggest that renal function may also influence the skeletal response to denosumab therapy, underlining the importance of personalized monitoring. In an Italian study conducted in postmenopausal osteoporotic women, renal function levels were associated with significant changes in bone mineral density during treatment with denosumab [63].
• Romosozumab: Approved by the FDA in April 2019, it is used to treat osteoporosis in postmenopausal women at a high risk of fractures. It functions by inhibiting sclerostin, both enhancing osteoblast and preventing osteoclast activity, and consequently promoting bone formation. Sclerostin does not only act on bone: it is also involved in vascular mechanisms, influencing vascular calcification and endothelial remodeling. Recent studies have highlighted its possible role as a mediator in cardiovascular physiopathology, suggesting a link between bone metabolism and vascular risk [64]. In chronic dialysis patients, osteoporosis remains inadequately managed despite the increased fracture risk. Romosozumab has been shown to increase bone mineral density in these patients without significant adverse effects, although hypocalcemia may occur, particularly when co-administered with calcium-sensing receptor agonists. This suggests that romosozumab may be a treatment option for severe osteoporosis in postmenopausal women on dialysis, but the careful monitoring of calcium levels is required. Its fracture prevention efficacy is evident in patients with moderate renal function (eGFR ≥ 30 mL/min/1.73 m^2^), while further studies are needed for those with more severe kidney impairment [65,66]. However, the use of romosozumab has raised concerns about a possible increase in cardiovascular risk, especially major ischemic events. Some clinical studies, such as the ARCH trial, have reported a higher incidence of myocardial infarction and stroke in patients treated with romosozumab compared to the control group. This has led regulatory agencies to contraindicate romosozumab in patients with previous major cardiovascular events. In light of this, the use of romosozumab in patients with CKD, already at high cardiovascular risk, must be carefully evaluated, favoring an individualized approach and a careful balance between skeletal benefits and cardiovascular risks [67].
• Calcium and Vitamin D Supplementation: If there are no biochemical signs of CKD-MBD (such as hyperparathyroidism or hyperphosphatemia), calcium and vitamin D intake should be similar to that of people without CKD. Daily calcium requirements vary depending on age and specific conditions. Taking calcium alone may slightly reduce fracture risk, primarily in older individuals. However, a more significant reduction is observed when calcium is combined with vitamin D [58]. The goal of vitamin D deficiency treatment is to rapidly restore normal serum levels of 25(OH)D. For severe deficiency, 50,000 IU of cholecalciferol weekly for 2–3 months is recommended, followed by maintenance up to 2000 IU daily. This aims to improve bone density, decrease fracture incidence, and improve the quality of life [58]. In recent years, extended-release calcifediol has received increasing attention as a therapeutic strategy in patients with CKD and hypovitaminosis D, particularly in those with secondary hyperparathyroidism. Unlike cholecalciferol, calcifediol bypasses hepatic hydroxylation and is more effective in rapidly normalizing 25(OH)D levels, with a predictable and more stable effect on PTH. In CKD stages G3–G4, the early use of calcifediol may prevent the progression of hyperparathyroidism and improve bone and cardiovascular outcomes. Furthermore, compared to calcitriol, which is the active form 1,25(OH)_2_D and acts rapidly but with a higher risk of hypercalcemia and vascular calcifications, calcifediol offers a more favorable safety profile, especially in the pre-dialysis phase. In addition to its well-known action on bone metabolism, vitamin D also exerts pleiotropic effects at the renal level, modulating tubular inflammation and contributing to the protection of tubular structure and function, as demonstrated in patients with tubulointerstitial nephropathies and chronic renal diseases [68]. A cost-effectiveness analysis evaluated early vitamin D therapy in CKD patients with vitamin D insufficiency and secondary hyperparathyroidism (SHPT) to prevent fractures and other complications [69]. Extended-release calcifediol has been shown to effectively increase 25-hydroxyvitamin D levels and reduce PTH levels in both clinical trials and real-world studies, regardless of CKD stage or baseline PTH levels. Initiating treatment in CKD stages G3 or G4, rather than waiting until stage G5/G5d, was found to be more cost-effective, reducing both cardiovascular events [70] and fractures [71].

### 7.3. Patients with eGFR < 30 mL/min/1.73 m^2^ (CKD Stages G4–G5 Non-Dialysis)

For patients with advanced CKD (eGFR < 30 mL/min/1.73 m^2^), osteoporosis management becomes more complex due to CKD-MBD. In these patients, therapeutic options must be cautiously selected, considering both efficacy and the risk of exacerbating metabolic disturbances. For this category of patients, it is crucial to rule out the presence of CKD-MBD through biochemical investigations or, if necessary, bone biopsy [7].

• Bisphosphonates are typically not recommended for patients with an eGFR below 30 mL/min/1.73 m^2^ (for alendronate, risedronate, and ibandronate) or below 35 mL/min/1.73 m^2^ (for zoledronic acid). Their routine use in patients with an eGFR < 30 mL/min/1.73 m^2^ is discouraged and should only be considered by clinicians specialized in CKD-MBD management, following comprehensive biochemical testing and/or bone biopsy to exclude ROD, especially in those with an eGFR < 15 mL/min/1.73 m^2^. In stage G4-G5 CKD patients, however, bisphosphonate use is typically avoided due to the risk of acute renal failure and atypical fractures [72].
• Denosumab: In advanced CKD, denosumab remains a viable option due to its non-renal clearance. Denosumab has been explored as a treatment option for bone loss in patients with CKD, particularly those with advanced stages [73]. Initial studies, such as the FREEDOM trial [74], demonstrated its efficacy in reducing fracture risk in osteoporotic women, with subsequent post hoc analyses showing similar benefits across varying levels of kidney function [75]. A retrospective study evaluated the effectiveness of denosumab in improving BMD in patients previously treated with bisphosphonates while also assessing the impact of CKD on treatment response. Among 134 patients, denosumab significantly increased BMD at the lumbar spine, total hip, and femoral neck. However, patients with an eGFR below 35 mL/min showed a lower response at the total hip and femoral neck compared to those with an eGFR above 35 mL/min, which was associated with PTH levels [76].

### 7.4. Patients on Hemodialysis (CKD Stage G5D)

As the eGFR declines, BMD decreases and fracture risk increases, with dialysis patients having the highest risk among CKD populations. For this population, fall prevention is critical as they are often frail and at a heightened risk of falling. Several factors contribute to this elevated fracture risk, including frailty, protein energy wasting (PEW), uremic toxins [77], diabetes, advanced glycation end products (AGEs), oxidative stress, and sarcopenia. Sarcopenia, characterized by reduced muscle mass and strength, further exacerbates their vulnerability. Key non-pharmacologic interventions, such as muscle strengthening and balance improvement, should be integrated into their management. Simple clinical tests, such as grip strength and gait speed, can help assess muscle weakness, while targeted physical therapy focused on core strength and balance training can reduce the risk of falls and fractures [78]. This phenomenon also affects patients undergoing PD [79].

• Non-Pharmacologic Measures: In addition to pharmacologic therapy, patients should follow non-pharmacologic strategies such a balanced diet, weight loss exercises [59], and fall prevention strategies. These measures are crucial for reducing fracture risk and supporting bone health, especially in patients with severe CKD. Moreover, it is recommended to aim for a total calcium intake (from diet and supplements) of 1200 mg/day, with no more than 500 mg/day coming from calcium supplements [58]. The remaining calcium should be obtained through dietary sources, such as calcium-fortified orange juice, soy products, and vegetables, especially for patients who need to limit dairy due to its phosphorus content. Additionally, a daily intake of 800 international units of vitamin D (either cholecalciferol or ergocalciferol) is suggested. In CKD-MBD patients with low BMD and fragility fractures, vitamin D supplementation can prevent secondary hyperparathyroidism, activate osteoblasts, relieve muscle weakness and myalgia, and reduce vascular calcification [80].
• The Management of Secondary Hyperparathyroidism: Controlling secondary hyperparathyroidism (SHPT) is critical for maintaining bone health in HD patients. Elevated PTH levels can lead to high bone turnover and increased fracture risk. Calcimimetics and vitamin D analogs are essential in managing secondary hyperparathyroidism and reducing bone resorption [81]. The efficacy of cinacalcet remains evident even in patients undergoing chronic HD with severe secondary hyperparathyroidism. Notably, improvements in bone markers, reductions in fibroblast growth factor 23 levels, and the stabilization of vascular calcification have been observed. Consequently, cinacalcet offers beneficial effects on chronic kidney disease-related mineral and bone disorder in severe cases of secondary hyperparathyroidism. It may serve as an effective initial therapy to lower PTH levels before considering surgical parathyroidectomy and as an alternative option for patients who are not suitable candidates for surgery [82]. Abnormalities in vitamin D metabolism are common, contributing to both bone fragility and vascular calcifications. Calcitriol, the active form of vitamin D, plays a key role in managing these imbalances. It was found that treatment with oral calcitriol significantly reduced the prevalence of vertebral fractures, without increasing the burden of aortic or iliac calcifications. This suggests that calcitriol may help mitigate fracture risk in CKD patients while maintaining a stable VC profile, though further research is needed to solidify these findings [83].

Etelcalcetide, a calcimimetic used to manage secondary hyperparathyroidism in HD patients, has demonstrated positive effects on bone health over a 36-week treatment period. In a recent study, etelcalcetide was associated with significant improvements in BMD and trabecular bone quality, specifically increasing areal BMD at key skeletal sites such as the spine, femoral neck, and total hip. Additionally, the TBS at the spine showed a notable improvement, reflecting better microarchitecture. Despite these positive changes, there was a reduction in bone turnover, as indicated by a decreased bone formation rate observed in biopsies. Importantly, these changes occurred without negatively affecting the material properties of the bone, suggesting that etelcalcetide may improve bone strength without compromising bone quality. These findings indicate that etelcalcetide could play a role in enhancing bone strength and reducing fracture risk in HD patients by improving both bone density and structural integrity. This makes it a potential therapeutic option in the management of osteoporosis and fracture prevention in this population [84].

• Denosumab in Hemodialysis: Denosumab has shown significant efficacy in improving BMD in patients with advanced CKD and those on dialysis (G5/G5D) [85]. Studies report progressive increases in both cortical and trabecular BMD over 2–3 years of treatment, particularly in key areas such as the hip region [86,87]. These improvements make denosumab a promising option for enhancing bone strength in this population, despite the lack of direct evidence for fracture risk reduction. However, the risk of hypocalcemia is a critical concern, especially in patients with CKD and those undergoing dialysis. Denosumab is neither metabolized nor excreted by the kidneys and is not dialyzable. Calcium and vitamin D supplementation are necessary during treatment to prevent severe hypocalcemia, a frequent complication in these patients due to their impaired renal function [86]. The close monitoring of calcium levels is essential to mitigate this risk and ensure the safety of denosumab therapy. Moreover, denosumab’s discontinuation poses additional challenges. Rapid and significant bone loss has been observed within one year after stopping treatment, particularly in the greater trochanter, highlighting the need for continued surveillance even after therapy cessation [88]. This underscores the importance of managing treatment carefully, especially considering the potential for BMD decline following discontinuation. While some authors support denosumab’s efficacy and safety in treating osteoporosis in HD patients, potentially reducing fracture risk, these risks—severe hypocalcemia during treatment and rapid bone loss after the discontinuation of treatment—should be considered with caution in clinical practice [89].
• Anabolic Therapy: Teriparatide, a recombinant 1-34 N-terminal sequence of human PTH, is an anabolic treatment for osteoporosis that may be cautiously used in patients with severe CKD. Although data on its safety are limited, recent studies indicate that teriparatide can effectively increase BMD and improve bone formation markers, even in patients undergoing dialysis. A Japanese post hoc analysis of elderly patients with advanced CKD demonstrated increased BMD and procollagen type 1 N-terminal propeptide (P1NP) levels without serious adverse events, highlighting its potential use in this high-risk group, though hypercalcemia needs careful monitoring [90]. Teriparatide may also be beneficial for patients with adynamic bone disease as it can inhibit sclerostin and promote bone formation. Although teriparatide has shown efficacy in improving BMD, it is contraindicated in those with a history of nephrolithiasis and can lead to adverse effects like hyperuricemia, hypercalcemia, and hypercalciuria. Compared to antiresorptive therapies like denosumab, which also improve BMD but carry a risk of severe hypocalcemia, teriparatide presents an anabolic alternative that could be particularly beneficial in CKD patients with osteoporosis. However, further large-scale studies are needed to confirm its long-term safety and effectiveness, especially in complex CKD–mineral and bone disorder cases [91].

Another anabolic option is abaloparatide, a synthetic analog of parathyroid hormone-related peptide(PTHrP[1–34]), approved for the treatment of postmenopausal osteoporosis at a high risk of fracture. It acts as a PTH1 receptor (PTH1R) agonist, stimulating osteoblastic activity and increasing bone mass. Compared to teriparatide, abaloparatide has a more selective effect on receptor activation, resulting in a lower incidence of hypercalcemia, potentially making it safer in patients with CKD. Clinical studies have shown a significant increase in bone mineral density and a reduction in the risk of vertebral fractures as early as 18 months of treatment. Although the AUC (area under the curve) increases in patients with renal insufficiency, no dosage adjustment is necessary, and efficacy has also been confirmed in subjects with moderately to severely reduced renal function. However, caution is required in patients with pre-existing hypercalcemia or a history of nephrolithiasis, and use is contraindicated in those at an increased risk of osteosarcoma (such as in rare metabolic bone diseases or previous bone radiotherapy). Pending specific studies in the dialysis population, abaloparatide represents a promising future alternative, especially for patients with CKD stages G3-G4 at a high risk of fracture and contraindications to vitamin D analogs or antiresorptive drugs [92].

## 8. Physical Activity for Bone Health in CKD

Physical activity and structured exercise interventions represent a cornerstone of the non-pharmacological strategy to maintain bone health and prevent fractures in patients with CKD, particularly those on dialysis. These interventions offer multidimensional benefits that extend beyond the musculoskeletal system by improving functional capacity, cardiorespiratory fitness, neuromuscular strength and the overall quality of life. Exercise programs are not only effective but also safe and feasible at all stages of CKD, including for dialysis patients and kidney transplant recipients, when appropriately adapted and monitored [93]. A systematic review on this topic reported that physical activity may be associated with improved BMD in both HD and kidney transplant recipients, although most evidence comes from cross-sectional studies [94]. However, interventional data provide encouraging results in patients with CKD in stages 3–4, HD and post transplant, emphasizing the potential of exercise to positively influence bone health [94].

Different exercise modalities have proven to have varying degrees of effectiveness. For example, aerobic exercise in patients with advanced CKD has been associated with a significant increase in circulating irisin and the attenuation of osteodystrophy progression. These effects appear to be due to the anabolic stimulation of osteoblasts via the integrin alphavbeta5 pathway, without causing any detectable burden on the kidneys [95]. Although evidence for BMD outcomes in dialysis patients is limited, in an experimental study included in the systematic review, no significant changes in BMD were observed in the lumbar spine or femoral neck after 12 weeks of intradialytic aerobic exercise training. Nevertheless, the BMD of the femoral neck was maintained in the intervention group compared to the control group, indicating a possible protective function [94]. Beyond skeletal endpoints, aerobic training remains essential for improving physical performance and mitigating cardiovascular risk [96,97].

Resistance training, performed either during dialysis treatment or independently, appears to have a more consistent effect on the bones. In HD and transplant patients, resistance training has shown improvements in BMD and bone turnover markers [94]. In a pilot study with older HD patients, a 24-week training program with traditional or cluster resistance training led to an increase in total BMD, femoral BMD, L3-L4 BMD and femoral neck BM [98]. In another study in which elastic bands and foot weights were used during intradialytic sessions, a significant increase in femoral neck BMD, an improvement in the T-score and a reduction in the prevalence of osteoporosis were observed after 24 weeks [99]. Despite a certain variability in the results in the different skeletal areas, resistance training consistently showed a positive effect, particularly on the femoral neck and proximal femur [94].

Combined aerobic and resistance programs also appear to be beneficial, although data on their specific effects on BMD are still lacking. Several meta-analyses and systematic reviews suggest that these protocols significantly improve the physical performance of dialysis patients. This is evidenced by improvements in 6 min walking distance, sitting and standing performance, grip strength and Short Physical Performance Battery scores [94,97]. These improvements indirectly lead to a reduced risk of falls—a major factor in fractures in the CKD population.

Balance training, which is often integrated into endurance-based programs, has shown particular promise in patients with CKD who do not yet require kidney replacement therapy. A 12-month randomized controlled trial showed that balance training combined with endurance training was superior to the combination of strength and endurance training in maintaining whole body BMD and improving T and Z scores in patients with stage 3–5 CKD [100].

The biochemical response to exercise in terms of bone turnover markers remains unclear. The above-mentioned systematic review reports that only a few markers—such as alkaline phosphatase (ALP), bone-specific ALP (BALP) and osteoprotegerin (OPG)—show significant modulation, while the levels of osteocalcin (OC), trap-5b and osteopontin (OPN) generally remain unchanged [2]. Observational studies suggest no clear association between physical activity and bone biomarkers in CKD cohorts [94]. However, exercise can stimulate osteoanabolic metabolic pathways—such as the canonical Wnt signaling cascade—and promote the release of protective bone-derived hormones such as Klotho and FGF23 [101,102]. Given the complexity of interpreting bone markers in CKD-MBD due to an altered mineral metabolism, further randomized trials are warranted [103].

A structured, multidisciplinary approach to physical activity in CKD is essential [93]. This includes personalized exercise prescriptions based on validated assessments such as the 6-Minute Walk Test (6MWT) and the Short Physical Performance Battery (SPPB), along with progressive training protocols lasting at least 12 weeks. Effective programs should integrate resistance, aerobic, and balance exercises. Nephrologists are encouraged to take an active role in prescribing physical activity and to collaborate with nurses, dietitians, physiotherapists, and exercise professionals. To support adherence, modern tools like wearable devices and virtual reality exergames are also recommended.

In addition to its physical and metabolic benefits, exercise is a key intervention for mitigating sarcopenia and protein energy wasting in individuals with chronic kidney disease, especially when paired with appropriate nutritional support. Resistance training, in particular, has demonstrated effectiveness even in the context of protein-restricted diets, promoting mitochondrial biogenesis and muscle hypertrophy [93].

The real-world implementation of exercise in CKD remains limited, hindered by several barriers. These include patient-related factors such as fatigue, the fear of injury, and comorbidities; staff-related challenges like insufficient training or limited time; and environmental constraints, including a lack of appropriate equipment or dedicated space for exercise interventions. Recognizing and addressing these obstacles is essential for integrating physical activity into routine CKD care [93]. The consensus recommends a stepwise approach to identifying and addressing barriers to exercise implementation through patient and staff education, interdisciplinary collaboration, and the integration of physical activity into routine care—particularly during dialysis sessions.

In conclusion, a growing body of evidence supports the use of individualized exercise programs, particularly those that include resistance and balance training, as safe, effective, and essential strategies for preserving bone health and physical function in individuals with CKD. Supported by national consensus and international guidelines, exercise should be considered a core component of comprehensive CKD management (Table 4).

**Table 4:** A summary of exercise modalities and their effects on bone health in patients with CKD. The table outlines the type of exercise, the main protocol characteristics (duration, frequency, and intensity), the CKD setting (pre-dialysis, dialysis, post-transplant), the observed outcomes in terms of BMD or related markers, and the corresponding references.

Exercise Type	Duration/Frequency/Intensity	CKD Stage/ Setting	Bone Outcomes	Key References
Aerobic training	30–45 min, 3×/week, moderate intensity	CKD stages 3–5, hemodialysis (HD), transplant recipients	Higher Irisin, potential osteoanabolic signaling; preserved femoral neck BMD	[94,95,96]
Resistance training	30–40 min, 2–3×/week, moderate-to-high intensity (e.g., cluster sets)	Mainly HD and post-transplant patients	↑ BMD (total, femoral, L3–L4), ↑ bone turnover markers	[97,98]
Combined (aerobic + resistance)	45–60 min, 2–3×/week, alternating modalities	Primarily HD; some data in CKD 3–4 and transplant	↑ Physical performance (6MWT, SPPB); indirect fall prevention	[93,96]
Balance training	20–30 min, 2×/week, progressive difficulty	CKD stages 3–5 (non-dialysis)	Preserved total BMD; ↑ T-score and Z-score	[99]

Notes: BMD = bone mineral density; CKD = chronic kidney disease; HD= hemodialysis. ↑: Higher.

## 9. Clinical Considerations

Despite increased awareness and the development of clinical guidelines, there remains a considerable unmet need for effective diagnostic and therapeutic strategies that address the complex interplay between CKD and bone health. Anti-resorptive therapies improve BMD and reduce fracture risk, although their safety and efficacy in advanced CKD are uncertain, due to potential side effects like hypocalcemia, particularly in later disease stages. Osteoanabolic agents, including teriparatide, may offer promise for patients with low bone turnover, specifically in advanced CKD, although more research is needed to establish their long-term safety. Parathyroidectomy is the last resort for severe renal hyperparathyroidism [82].

The evolution of KDIGO guidelines on CKD-MBD reflects the growing understanding of the complex pathophysiology involved in bone disorders associated with CKD. The initial 2006 KDIGO position statement established a foundational framework for defining and classifying ROD [16], which was expanded in 2009 to include specific diagnostic and management recommendations. The 2017 update introduced substantial changes, integrating new evidence on treatments like non-calcium-based phosphate binders and calcimimetics, emphasizing an individualized approach to treatment based on CKD stage and patient-specific factors [42].

Moreover, the 2017 KDIGO guidelines [7,44], marked a significant shift by recommending BMD assessment for CKD patients at a risk of fractures, a departure from earlier recommendations. This change emphasizes the growing recognition of osteoporosis as a critical issue in CKD management. However, the applicability of traditional diagnostic tools like DXA in CKD patients remains debated. DXA, while valuable in estimating BMD, may not fully capture the nuances of BQ that are affected by CKD, such as changes in microarchitecture and bone turnover [16,46].

In terms of therapeutic strategies, this review has highlighted the complexity of managing osteoporosis in patients with CKD, particularly in advanced stages. Pharmacological interventions, such as bisphosphonates and denosumab, have been extensively studied in the general population with promising results but their use in CKD patients requires cautious consideration [3]. Denosumab, for example, looks promising because of its renal clearance-independent mechanism, but the risk of hypocalcemia in advanced stages of CKD remains a major concern.

Anabolic agents, such as teriparatide, may offer valuable options, particularly in adynamic bone disease in which low bone turnover is a major problem. However, further research is required to determine the long-term safety and efficacy of these treatments in the CKD population [10].

Despite these advances, there is still limited consensus on optimal management strategies, particularly in the context of individualized treatment based on CKD stage and patient-specific factors. The KDIGO guidelines provide a useful framework, but as this review has highlighted, more research is needed to tailor treatments to specific patient populations. Furthermore, a multidisciplinary approach involving nephrologists, endocrinologists, and bone specialists is essential to optimize patient care and address the multifactorial nature of CKD-MBD [Table 5].

A critical limitation of the current literature lies in the limited inclusion of CKD stage G4-G5D patients in pivotal osteoporosis trials. Many randomized controlled studies evaluating bisphosphonates, denosumab, and newer agents—such as romosozumab and abaloparatide—either excluded individuals with impaired renal function or failed to stratify outcomes by CKD stage. This exclusion substantially limits the generalizability of findings to the nephrology population. As a result, much of the therapeutic evidence cited in this review is derived from studies conducted in the general population or in patients with only mild-to-moderate CKD. Clinicians should interpret these data cautiously, particularly when managing patients on dialysis or with advanced renal dysfunction. There remains a critical need for prospective trials specifically designed to assess the efficacy and safety of osteoporosis treatments in patients with CKD G4-G5D.

## 10. Conclusions

This review highlights the significant large burden of fractures and bone fragility in CKD patients, particularly in the advanced stages of the disease. Although progress has been made in understanding the pathophysiology and management of CKD-MBD, significant knowledge gaps remain. Tailoring diagnostic and therapeutic approaches to meet the specific needs of CKD patients is essential for improving clinical outcomes. Future research should focus on developing more accurate tools for assessing fracture risk in these patients and exploring the long-term safety of novel therapeutic agents.

Given the profound impact of fractures on patient-centered outcomes, additional clinical studies are necessary to address these high-priority needs. Multifactorial clinical assessment tools are urgently needed to estimate fracture risk in the context of CKD-MBD more precisely. Favorable epidemiological evidence suggests that high accuracy in predicting fractures in dialysis patients and individualized drug therapy, including dietary supplements and bone-active agents, is essential in managing these risks. However, existing therapeutic strategies often overlook nephropathy-related bone complications. This gap can be addressed by incorporating the 2017 KDIGO Clinical Practice Guidelines, which advocate personalized treatment based on eGFR and bone metabolism status.

The effective management of osteoporosis in CKD patients requires a multidisciplinary approach, involving nephrologists, endocrinologists, and orthopedic specialists, to ensure the comprehensive care of both renal and skeletal health. The KDIGO guidelines recommend regular BMD assessments and individualized therapeutic strategies to mitigate fracture risk, further emphasizing the need for collaborative, patient-centered care.

## Figures and Tables

**Table 1:** Underlying mechanisms of bone fractures in CKD.

Mechanism	Pathophysiological Features	Clinical Implications
Mineral Metabolism Imbalance	Impaired P excretion and ↓ Ca reabsorption due to declining renal function.	Predisposes individuals to osteomalacia and poor bone quality; necessitates correction of the imbalance.
Secondary Hyperparathyroidism	Higher PTH levels increase osteoclastic activity, leading to excessive bone resorption and lower strength.	Associated with osteitis fibrosa cystica; requires PTH monitoring and control.
Vitamin D deficiency	↓ synthesis of 1,25(OH)2D due to loss of 1-α-hydroxylaze activity leads to ↓ Ca absorption and impaired bone formation.	↑ susceptibility to fractures; requires vitamin D replacement and monitoring.
Uremic toxin accumulation	Retention of toxins like indoxyl sulfate and AGEs inhibits osteoblast function and enhances osteoclast activity.	Linked to skeletal resistance to PTH and reduced bone quality; highlights need for toxin reduction strategies.
Bone remodeling disruption	Disorganized or suppressed bone turnover, often due to suppressed PTH, leads to structurally weak bone.	Associated with adynamic bone disease and fracture risk; necessitates tailored treatment decisions.
Vascular calcification	Deposition of calcium in blood vessels limits calcium availability for bone.	Increases risk of cardiovascular events and skeletal fragility; complicates treatment planning.
Inflammation and oxidative stress	Chronic inflammation and ROS damage osteocyte and impair bone healing, contributing to cortical porosity.	Exacerbates bone loss and increases fracture risk; anti-inflammatory interventions may be beneficial.
Sarcopenia and muscle weakness	Reduced muscle mass and strength increase fall risk and lead to reduced mechanical loading on bone, weakening bone structure.	Important contributor to falls and fracture risk in older CKD patients; requires exercise and nutrition intervention.

Notes: AGEs = advanced glycation end-products; CKD = chronic kidney disease; PTH = parathyroid hormone; ROS = reactive oxygen species. ↑: Higher; ↓: lower.

**Table 2:** Diagnostic management options in CKD-related bone fragility.

Diagnostic Tool	Purpose	CKD-Specific Considerations
Serum Biochemical Markers	Calcium, phosphate, vitamin D, and FGF-23 levels are useful to understand mineral metabolism.	Interpretation must account for CKD stage and concurrent therapies.
PTH	Reflects bone turnover status; essential in distinguishing high vs. low turnover disease.	PTH targets are CKD stage-specific; extreme values indicate altered turnover.
BSAP	Marker of osteoblastic activity; helps evaluate bone formation.	May be elevated in high turnover states; needs correlation with other markers.
BMD through DXA	Quantifies bone mineral content; commonly used for osteoporosis diagnosis.	May overestimate bone strength due to vascular calcifications or soft tissue changes
TBS	Provides information on bone microarchitecture and fracture risk beyond BMD.	Complementary to DXA; useful in CKD stages where BMD is less predictive.
FRAX, Adapted	Estimates fracture probability, though not validated for CKD (requires clinical adaptation).	Not fully validated in CKD; clinical judgment required to adjust risk estimates.
Vascular Imaging (e.g., CT, X-ray)	Identifies vascular calcifications often associated with CKD-MBD.	Helpful in advanced disease; calcifications may influence treatment choices.
Bone Biopsy	Gold standard for diagnosing renal osteodystrophy and differentiating bone turnover types.	Invasive and rarely used clinically; reserved for complex cases or research.

Notes: BMD = bone mineral density; BSAP= bone-specific alkaline phosphatase; CKD = chronic kidney disease; CT = computed tomography; DXA= dual-energy X-ray absorptiometry; FGF23 = fibroblast growth factor 23; FRAX = fracture risk assessment tool; MBD = mineral bone disorder; TBS = trabecular bone score.

**Table 3:** Therapeutic strategies for bone fragility in chronic kidney disease by CKD stage.

CKD Stages	Drug	Mechanism	Pros and Cons	Key Considerations
All	Calcium and vitamin D (cholecalciferol, calcitriol)	Supports bone mineralization, suppresses PTH	Essential for bone health; risk of hypercalcemia and vascular calcifications	Monitor levels, avoid overload
G1-G3 (eGFR ≥ 30 mL/min/1.73 m^2^)	Biphosphonates (alendronate, risedronate)	Inhibit osteoclast-mediated bone resorption	Pros: improve BMD, reduce fracture risk; cons: risk of renal complications, gastrointestinal side effects	Avoid in severe CKD, monitor renal function
	Denosumab	RANKL inhibitor, reduces osteoclast activity	Pros: not renally cleared, improves BMD; cons: risk of hypocalcemia, rebound bone loss after discontinuation	Monitor calcium, continue supplementation
	Romosozumab	Sclerostin inhibitor, promotes bone formation	Pros: increases BMD, reduces fractures; cons: potential cardiovascular risk	Avoid in patients with cardiovascular disease history
G4-G5 (eGFR < 30 mL/min/1.73 m^2^)	Denosumab	RANKL inhibitor	Pros: effective in advanced CKD; cons: higher hypocalcemia risk	Close calcium monitoring required
G5D (dialysis)	Cinacalcet, etelcalcetide	Calcimimetics, decrease PTH	Pros: control secondary hyperparathyroidism; cons: may reduce bone turnover	Monitor bone biomarkers, adjust dose
	Teriparatide	Anabolic, stimulates osteoblasts	Pros: improves BMD and bone markers; cons: hypercalcemia, contraindicated in nephrolithiasis	Cautious use, monitor closely
G3-G5	Extended-release calcifediol	Vitamin D prohormone, increases 25 (OH)D and suppresses PTH	Pros: effective in SHPT, fewer side effects; cons: less effective in late CKD	Use early in CKD; monitor 25(OH)D and PTH

Notes: BMD = bone mineral density; CKD = chronic kidney disease; eGFR = estimated glomerular filtration rate; PTH = parathyroid hormone; RANKL = Receptor Activator of Nuclear Factor Kappa-B Ligand; SHPT = secondary hyperparathyroidism.

**Table 5:** This table outlines a structured approach to osteoporosis in chronic kidney disease, summarizing key elements across epidemiology, pathophysiology, diagnostics, treatment strategies, and special considerations. Chronic kidney disease (CKD), dual-energy X-ray absorptiometry (DXA), the Fracture Risk Assessment Tool (FRAX), high-resolution peripheral quantitative computed tomography (HR-pQCT).

Category	Practical Points	Details
Epidemiology and Risk	High fracture risk in CKD	CKD G3–G5D patients have 2.5 increased fracture risk. Hip fractures are particularly morbid.
	Osteoporosis is prevalent in CKD	18–32% prevalence in CKD; especially common in G4–G5 and dialysis patients.
Pathophysiology	CKD-MBD overlap	Includes phosphate retention, low vitamin D, secondary hyperparathyroidism, and vascular calcifications.
	Bone quality degradation	Low turnover diseases (e.g., adynamic bone), osteitis fibrosa, and osteomalacia all contribute.
	Vitamin K deficiency	Leads to vascular calcification and bone fragility.
	Sarcopenia impact	Muscle wasting in CKD increases fall and fracture risk, especially in elderly.
Diagnosis	BMD (via DXA)	Recommended in CKD G3–G5D with fracture risk factors per 2017 KDIGO. Interpret cautiously in G4–G5D due to vascular calcification.
	Trabecular bone score (TBS)	Additive to DXA; may identify fragility not detected by BMD alone.
	Biomarkers	Assess PTH, calcium, phosphate, BSAP, 25(OH)D, and FGF23 to understand turnover type.
	Risk tools	Use FRAX/FRAXplus, DeFRA, and K-DeFRA to estimate risk.
	HR-pQCT/QUS	Consider where available for advanced structural insight.
Treatment Strategy	Multidisciplinary care	Include nephrologists, endocrinologists, rheumatologists, geriatricians.
	Treat based on turnover status	High turnover: avoid anabolic agents; low turnover: avoid antiresorptives.
	Calcium and vitamin D	Supplement carefully to avoid vascular calcification. Use active vitamin D in later CKD.
	Antiresorptive therapy	Denosumab may be used, even in dialysis; monitor for hypocalcemia. Bisphosphonates are controversial in G4–G5D.
	Anabolic therapy	Teriparatide or romosozumab may be considered in low turnover disease; use with caution and specialist input.
	Non-pharmacologic measures	Fall prevention, resistance exercise, sarcopenia management, smoking/alcohol cessation.
Special Considerations	Dialysis patients	Higher mortality post fracture; consider tailored tools (e.g., K-DeFRA).
	FRAX limitations	May underestimate or overestimate fracture risk; combine with biomarkers and clinical judgment.
	Biopsy/histomorphometry	Gold standard for turnover diagnosis but rarely performed. Reserved for unclear or refractory cases.

## Data Availability

No new data were created or analyzed in this study.

## References

[1] Jørgensen H.S., Lloret M.J., Lalayiannis A.D., Shroff  R., Evenepoel P. (2024). European Renal Osteodystrophy (EUROD) initiative of the CKD-MBD working group of the European Renal Association (ERA), and the CKD-MBD and Dialysis working groups of the European Society of Pediatric Nephrology. Ten tips on how to assess bone health in patients with chronic kidney disease. Clin. Kidney J..

[2] Hsu C.Y., Chen L.R., Chen K.H. (2020). Osteoporosis in Patients with Chronic Kidney Diseases: A Systemic Review. Int. J. Mol. Sci..

[3] Bover J., Bailone L., López-Báez V. (2017). Osteoporosis, bone mineral density and CKD-MBD: Treatment considerations. J. Nephrol..

[4] McNerny E.M.B., Nickolas T.L. (2017). Bone Quality in Chronic Kidney Disease: Definitions and Diagnostics. Curr. Osteoporos. Rep..

[5] Fusaro M., Aghi A., Mereu M.C., Giusti A. (2017). Fratture da fragilità nella Malattia Renale Cronica (MRC) [Fragility fracture in the Chronic Kidney Disease (CKD)]. G. Ital. Nefrol..

[6] Iseri K., Carrero J.J., Evans M. (2020). Incidence of Fractures Before and After Dialysis Initiation. J. Bone Miner. Res..

[7] Kidney Disease: Improving Global Outcomes (KDIGO) CKD-MBD Update Work Group (2017). KDIGO 2017 Clinical Practice Guideline Update for the Diagnosis, Evaluation, Prevention, and Treatment of Chronic Kidney Disease-Mineral and Bone Disorder (CKD-MBD). Kidney Int. Suppl..

[8] Pazianas M., Miller P.D. (2021). Osteoporosis and Chronic Kidney Disease-Mineral and Bone Disorder (CKD-MBD): Back to Basics. Am. J. Kidney Dis..

[9] Francis A., Harhay M.N., Ong A.C.M. (2024). American Society of Nephrology; European Renal Association; International Society of Nephrology. Chronic kidney disease and the global public health agenda: An international consensus. Nat. Rev. Nephrol..

[10] Ziolkowski S., Liu S., Montez-Rath M.E., Denburg M., Winkelmayer W.C., Chertow G., O’Shaughnessy M.M. (2022). Association between cause of kidney failure and fracture incidence in a national US dialysis population cohort study. Clin. Kidney J..

[11] Kanis J.A., Odén A., McCloskey E.V. (2012). A systematic review of hip fracture incidence and probability of fracture worldwide. Osteoporos. Int..

[12] Ballane G., Cauley J.A., Luckey M.M., El-Hajj Fuleihan G. (2017). Worldwide prevalence and incidence of osteoporotic vertebral fractures. Osteoporos. Int..

[13] Sessa C., Galeano D., Alessandrello I., Aprile G., Distefano G., Ficara V., Giglio E., Musumeci S., Pocorobba B., Zuppardo C. (2019). Osteoporosis and chronic kidney disease: Review and new therapeutic strategies. G. Ital. Nefrol..

[14] Haarhaus M., Aaltonen L., Cejka D., Cozzolino M., de Jong  R.T., D’Haese P., Evenepoel  P., Lafage-Proust M.H., Mazzaferro S., McCloskey  E. (2022). Management of fracture risk in CKD-traditional and novel approaches. Clin. Kidney J..

[15] NIH Consensus Development Panel on Osteoporosis Prevention, Diagnosis, and Therapy (2001). Osteoporosis prevention, diagnosis, and therapy. JAMA.

[16] Moe S., Drüeke T., Cunningham J., Goodman W., Martin K., Olgaard K., Ott S., Sprague S., Lameire N., Eknoyan G. (2006). Kidney Disease: Improving Global Outcomes (KDIGO). Definition, evaluation, and classification of renal osteodystrophy: A position statement from Kidney Disease: Improving Global Outcomes (KDIGO). Kidney Int..

[17] David V., Salusky I.B., Malluche H., Nickolas T.L. (2023). Renal osteodystrophy: Something old, something new, something needed. Curr. Opin. Nephrol. Hypertens..

[18] Carbonara C.E.M., Reis L.M.D., Quadros K.R.D.S., Vieira Roza  N.A., Sano R., Carvalho A.B., Jorgetti V., de Oliveira R.B. (2020). Renal osteodystrophy and clinical outcomes: Data from the Brazilian Registry of Bone Biopsies—REBRABO. J. Bras. Nefrol..

[19] Jha S., Chapman M., Roszko K. (2019). When Low Bone Mineral Density and Fractures Is Not Osteoporosis. Curr. Osteoporos. Rep..

[20] Cohen-Solal M., Funck-Brentano T., Ureña Torres P. (2020). Bone fragility in patients with chronic kidney disease. Endocr. Connect..

[21] Ong T., Yong B.K.A., Shouter T., Shahrokhi N., Sahota O. (2020). Optimising bone health among older people with hip fractures and co-existing advanced chronic kidney disease. Eur. Geriatr. Med..

[22] Keung L., Perwad F. (2018). Vitamin D and kidney disease. Bone Rep..

[23] Aguilar A., Gifre L., Ureña-Torres P., Carrillo-López  N., Rodriguez-García M., Massó E., da Silva I., López-Báez V., Sánchez-Bayá M., Prior-Español A. (2023). Pathophysiology of bone disease in chronic kidney disease: From basics to renal osteodystrophy and osteoporosis. Front. Physiol..

[24] Sessa C., Granata A., Gaudio A., Xourafa A., Malatino L., Lentini P., Fatuzzo P., Rapisarda F., Castellino P., Zanoli L. (2020). Vascular dysfunction in Cardiorenal Syndrome type 4. G. Ital. Nefrol..

[25] Bellone F., Cinquegrani M., Nicotera R., Carullo N., Casarella A., Presta P., Andreucci M., Squadrito G., Mandraffino G., Prunesti M. (2022). Role of Vitamin K in Chronic Kidney Disease: A Focus on Bone and Cardiovascular Health. Int. J. Mol. Sci..

[26] Sabatino A., Cuppari L., Stenvinkel Pet a.l., Lindholm B., Avesani C.M. (2021). Sarcopenia in chronic kidney disease: What have we learned so far?. J. Nephrol..

[27] Fusaro M., Pereira L., Bover J. (2023). Current and Emerging Markers and Tools Used in the Diagnosis and Management of Chronic Kidney Disease-Mineral and Bone Disorder in Non-Dialysis Adult Patients. J. Clin. Med..

[28] Vervloet M.G., Brandenburg V.M., CKD-MBD working group of ERA-EDTA (2017). Circulating markers of bone turnover. J. Nephrol..

[29] Bover J., Ureña-Torres P., Cozzolino M., Rodríguez-García M., Gómez-Alonso C. (2021). The Non-invasive Diagnosis of Bone Disorders in CKD. Calcif. Tissue Int..

[30] Ensrud K.E., Crandall C.J. (2024). Osteoporosis. Ann. Intern. Med..

[31] Catalano A., Morabito N., Basile G., Fusco S., Castagna G., Reitano F., Albanese R.C., Lasco A. (2013). Fracture risk assessment in postmenopausal women referred to an Italian center for osteoporosis: A single day experience in Messina. Clin. Cases Miner. Bone Metab..

[32] Przedlacki J., Buczyńska-Chyl J., Koźmiński P., Niemczyk E., Wojtaszek E., Gieglis  E., Żebrowski  P., Podgórzak  A., Wściślak J., Wieliczko M. (2020). FRAX prognostic and intervention thresholds in the management of major bone fractures in hemodialysis patients: A two-year prospective multicenter cohort study. Bone.

[33] Jafari M., Anwar S., Kour K., Sanjoy S., Goyal K., Prasad B. (2021). T Scores, FRAX, Frailty Phenotype, Falls, and Its Relationship to Fractures in Patients on Maintenance Hemodialysis. Can. J. Kidney Health Dis..

[34] Tan T.H.A., Johansson H., Harvey N.C., Lorentzon M., Kanis J.A., McCloskey E., Schini M. (2024). Assessment of fracture risk with FRAX and FRAXplus. Gac. Med. Mex..

[35] Adami S., Bianchi G., Brandi M.L., Di Munno O., Frediani B., Gatti D., Giannini S., Girasole G., Minosola G., Minosola S. (2010). Validation and further development of the WHO 10-year fracture risk assessment tool in Italian postmenopausal women: Project rationale and description. Clin. Exp. Rheumatol..

[36] Catalano A., Gaudio A., Bellone F., La Fauci M.M., Xourafa A., Gembillo G., Basile G., Natale G., Squadrito G., Corica F. (2022). Trabecular bone score and phalangeal quantitative ultrasound are associated with muscle strength and fracture risk in hemodialysis patients. Front. Endocrinol..

[37] Pimentel A., Bover J., Elder  G., Cohen-Solal  M., Ureña-Torres P.A. (2021). The Use of Imaging Techniques in Chronic Kidney Disease-Mineral and Bone Disorders (CKD-MBD)-A Systematic Review. Diagnostics.

[38] Lim C.Y., Ong K.O. (2013). Various musculoskeletal manifestations of chronic renal insufficiency. Clin. Radiol..

[39] Olsen K.M., Chew F.S. (2006). Tumoral calcinosis: Pearls, polemics, and alternative possibilities. Radiographics.

[40] Link T.M. (2012). Osteoporosis imaging: State of the art and advanced imaging. Radiology.

[41] Chen M., Gerges M., Raynor W.Y., Uk Park P.S., Nguyen E., Chan D.H., Gholamrezanezhad A. (2024). State of the Art Imaging of Osteoporosis. Semin. Nucl. Med..

[42] (2009). Kidney Disease: Improving Global Outcomes (KDIGO) CKD-MBD Work Group. KDIGO clinical practice guideline for the diagnosis, evaluation, prevention, and treatment of Chronic Kidney Disease-Mineral and Bone Disorder (CKD-MBD). Kidney Int. Suppl..

[43] Cailleaux P.E., Ostertag A., Metzger M., Stengel B., Boucquemont J., Houillier P., Flamant M., Ureña-Torres P., Cohen-Solal M. (2021). NephroTest Study group. Longitudinal Bone Loss Occurs at the Radius in CKD. Kidney Int. Rep..

[44] Ketteler M., Block G.A., Evenepoel P., Fukagawa M., Herzog C.A., McCann L., Moe S.M., Shroff R., Tonelli M.A., Toussaint N.D. (2017). Executive summary of the 2017 KDIGO Chronic Kidney Disease-Mineral and Bone Disorder (CKD-MBD) Guideline Update: What’s changed and why it matters. Kidney Int..

[45] Bover J., Gómez-Alonso C., Casado E., Rodríguez-García M., Lloret M.J., Castro-Alonso C., Gifre L., Henríquez-Palop F., Prior-Español  A., de la Manzanara V.L. (2024). Osteoporosis management in patients with chronic kidney disease (ERCOS Study): A challenge in nephrological care. Nefrologia.

[46] Prasad B., Ferguson T., Tangri N., Yong Ng C., Nickolas T.L. (2019). Association of Bone Mineral Density with Fractures Across the Spectrum of Chronic Kidney Disease: The Regina CKD-MBD Study. Can. J. Kidney Health Dis..

[47] Jones B.C., Lee H., Cheng C.C.,  Al Mukaddam M.,  Song H.K., Snyder  P.J., Kamona N., Rajapakse C.,  Wehrli F.W. (2023). MRI Quantification of Cortical Bone Porosity, Mineralization, and Morphologic Structure in Postmenopausal Osteoporosis. Radiology.

[48] Bae W.C. (2023). Advances and Shortfalls in MRI Evaluation of Osteoporosis. Radiology.

[49] Moayyeri A., Adams J.E., Adler R.A., Krieg M.A., Hans D., Compston J., Lewiecki E.M. (2012). Quantitative ultrasound of the heel and fracture risk assessment: An updated meta-analysis. Osteoporos. Int..

[50] Wittich A., Vega E., Casco C., Marini A., Forlano C., Segovia F., Nadal M., Mautalen C. (1998). Ultrasound velocity of the tibia in patients on hemodialysis. J. Clin. Densitom..

[51] Park P.S.U., Werner T.J., Alavi A. (2024). PET/CT for the Opportunistic Screening of Osteoporosis and Fractures in Cancer Patients. Curr. Osteoporos. Rep..

[52] Uchida K., Nakajima H., Miyazaki T., Yayama T., Kawahara H., Kobayashi S., Tsuchida T., Okazawa H., Fujibayashi Y., Baba H. (2009). Effects of alendronate on bone metabolism in glucocorticoid-induced osteoporosis measured by 18F-fluoride PET: A prospective study. J. Nucl. Med..

[53] Evenepoel P., D’Haese P., Bacchetta J., Cannata-Andia J., Ferreira A., Haarhaus M., Mazzaferro S., Proust M.H.L., Salam S., Spasovski G. (2017). Bone biopsy practice patterns across Europe: The European renal osteodystrophy initiative-a position paper. Nephrol. Dial. Transplant..

[54] Beto J.A. (2015). The role of calcium in human aging. Clin. Nutr. Res..

[55] Heaney R.P., Dowell M.S., Barger-Lux M.J. (1999). Absorption of calcium as the carbonate and citrate salts, with some observations on method. Osteoporos. Int..

[56] Esche J., Johner S., Shi L.,  Schönau E., Remer T. (2016). Urinary Citrate, an Index of Acid-Base Status, Predicts Bone Strength in Youths and Fracture Risk in Adult Females. J. Clin. Endocrinol. Metab..

[57] Kommer A., Kostev K., Schleicher E.M., Weinmann-Menke  J., Labenz C. (2024). Proton pump inhibitor use and bone fractures in patients with chronic kidney disease. Nephrol. Dial. Transplant..

[58] Kanis J.A., Cooper C., Rizzoli R., Reginster  J.Y. (2019). Scientific Advisory Board of the European Society for Clinical and Economic Aspects of Osteoporosis and Osteoarthritis (ESCEO) and the Committees of Scientific Advisors and National Societies of the International Osteoporosis Foundation (IOF). Executive summary of European guidance for the diagnosis and management of osteoporosis in postmenopausal women. Aging Clin. Exp. Res..

[59] Jørgensen H.S., David K., Salam S., Evenepoel 6 P. (2021). Traditional and Non-traditional Risk Factors for Osteoporosis in CKD. Calcif. Tissue Int..

[60] Castro-Alonso C., D’Marco L., Pomes J., Del Amo Conill M., García-Diez A.I., Molina P., Puchades M.J., Valdivielso J.M., Escudero  V., Bover J. (2020). Prevalence of Vertebral Fractures and Their Prognostic Significance in the Survival in Patients with Chronic Kidney Disease Stages 3–5 Not on Dialysis. J. Clin. Med..

[61] Alarkawi D., Ali M.S., Bliuc D., Pallares N., Tebe C., Elhussein L., Caskey F.J., Arden N.K., Ben-Shlomo Y., Abrahamsen B. (2020). Oral Bisphosphonate Use and All-Cause Mortality in Patients with Moderate-Severe (Grade 3B-5D) Chronic Kidney Disease: A Population-Based Cohort Study. J. Bone Miner. Res..

[62] Iseri K., Mizobuchi M., Winzenrieth R., Humbert L., Saitou T., Kato T., Nakajima Y., Wakasa M., Shishido K., Honda H. (2023). Long-Term Effect of Denosumab on Bone Disease in Patients with CKD. Clin. J. Am. Soc. Nephrol..

[63] Catalano A., Oliveri C., Natale G., Agostino R.M., Squadrito G., Gaudio A., Gembillo G., Marina D., Cernaro V., Longhitano E. (2024). Renal Function Is Associated with Changes in Bone Mineral Density in Postmenopausal Osteoporotic Women Treated with Denosumab: Data from a Retrospective Cohort Study. J. Clin. Med..

[64] Catalano A., Bellone F., Morabito N., Corica F. (2020). Sclerostin and Vascular Pathophysiology. Int. J. Mol. Sci..

[65] Miyauchi A., Hamaya E., Nishi K., Tolman C., Shimauchi J. (2022). Efficacy and safety of romosozumab among Japanese postmenopausal women with osteoporosis and mild-to-moderate chronic kidney disease. J. Bone Miner. Metab..

[66] Saito T., Mizobuchi M., Kato T., Suzuki T., Fujiwara Y., Kanamori N., Makuuchi M., Honda H. (2023). One-Year Romosozumab Treatment Followed by One-Year Denosumab Treatment for Osteoporosis in Patients on Hemodialysis: An Observational Study. Calcif. Tissue Int..

[67] Shoback D., Rosen C.J., Black D.M., Cheung A.M., Murad H., Eastell R. (2020). Pharmacological Management of Osteoporosis in Postmenopausal Women: An Endocrine Society Guideline Update. J. Clin. Endocrinol. Metab..

[68] Gembillo G., Cernaro V., Siligato R.,  Curreri F., Catalano A., Santoro D. (2020). Protective Role of Vitamin D in Renal Tubulopathies. Metabolites.

[69] Snyder S., Hollenbeak C.S., Kalantar-Zadeh K., Gitlin M., Ashfaq A. (2020). Cost-Effectiveness and Estimated Health Benefits of Treating Patients with Vitamin D in Pre-Dialysis. Forum Health Econ. Policy.

[70] Brandenburg V., Ketteler M. (2022). Vitamin D and Secondary Hyperparathyroidism in Chronic Kidney Disease: A Critical Appraisal of the Past, Present, and the Future. Nutrients.

[71] Merante D., Schou H., Morin I., Manu M., Ashfaq A., Bishop C., Strugnell S. (2024). Extended-Release Calcifediol: A Data Journey from Phase 3 Studies to Real-World Evidence Highlights the Importance of Early Treatment of Secondary Hyperparathyroidism. Nephron.

[72] Abrahamsen B., Ernst M.T., Smith C.D., Nybo M., Rubin K.H., Prieto-Alhambra D., Hermann A.P. (2020). The association between renal function and BMD response to bisphosphonate treatment: Real-world cohort study using linked national registers. Bone.

[73] Gopaul A., Kanagalingam T., Thain J., Khan T., Cowan A., Sultan N., Clemens K. (2021). Denosumab in chronic kidney disease: A narrative review of treatment efficacy and safety. Arch. Osteoporos..

[74] Kendler D.L., Chines A., Brandi M.L., Papapoulos S., Lewiecki E.M., Reginster J.Y., Torres  M.M., Wang A., Bone H.G. (2019). The risk of subsequent osteoporotic fractures is decreased in subjects experiencing fracture while on denosumab: Results from the FREEDOM and FREEDOM Extension studies. Osteoporos. Int..

[75] Gronskaya S., Belaya Z., Rozhinskaya L., Mamedova E., Vorontsova M., Solodovnikov S., Golounina O., Melnichenko G. (2023). Denosumab for osteoporosis in patients with primary hyperparathyroidism and mild-to-moderate renal insufficiency. Endocrine.

[76] Fraser T.R., Flogaitis I., Moore A.E., Hampson G. (2020). The effect of previous treatment with bisphosphonate and renal impairment on the response to denosumab in osteoporosis: A ‘real-life’ study. J. Endocrinol. Investig..

[77] Yamamoto S., Fukagawa M. (2017). Uremic Toxicity and Bone in CKD. J. Nephrol..

[78] Papakonstantinopoulou K., Sofianos I. (2017). Risk of falls in chronic kidney disease. J. Frailty Sarcopenia Falls..

[79] Davenport A. (2022). Frailty, appendicular lean mass, osteoporosis and osteosarcopenia in peritoneal dialysis patients. J. Nephrol..

[80] Shen Y. (2023). Role of nutritional vitamin D in chronic kidney disease-mineral and bone disorder: A narrative review. Medicine.

[81] Salam S.N., Khwaja A., Wilkie M.E. (2016). Pharmacological Management of Secondary Hyperparathyroidism in Patients with Chronic Kidney Disease. Drugs.

[82] Susantitaphong P., Vadcharavivad S., Susomboon T., Singhan W., Dumrongpisutikul  N., Jakchairoongruang K., Eiam-Ong S., Praditpornsilpa K. (2019). The effectiveness of cinacalcet: A randomized, open label study in chronic hemodialysis patients with severe secondary hyperparathyroidism. Ren. Fail..

[83] Fusaro M., Cianciolo G., Tripepi G., Plebani M., Aghi A., Politi C., Zaninotto M., Nickolas T.L., Ferrari S., Ketteler M. (2021). Oral Calcitriol Use, Vertebral Fractures, and Vitamin K in Hemodialysis Patients: A Cross-Sectional Study. J. Bone Miner. Res..

[84] Khairallah P., Cherasard J., Sung J., Agarwal S., Aponte M.S., Bucovsky M., Fusaro M., Silberzweig J., Frumkin G.N., El Hachem K. (2023). Changes in Bone Quality after Treatment with Etelcalcetide. Clin. J. Am. Soc. Nephrol..

[85] Hori M., Yasuda K., Takahashi H., Kondo  C., Shirasawa Y., Ishimaru Y., Sekiya Y., Morozumi K., Maruyama S. (2022). Effects of bone turnover status on the efficacy and safety of denosumab among haemodialysis patients. Sci. Rep..

[86] Hsu C.T., Deng Y.L., Chung M.C., Tsai S.F., Lin S.Y., Chen C.H. (2023). Integrated Osteoporosis Care to Reduce Denosumab-Associated Hypocalcemia for Patients with Advanced Chronic Kidney Disease and End-Stage Renal Disease. Healthcare.

[87] Kim H., Lee E.J., Woo S., Rho S., Jung J.Y. (2024). Effect of Denosumab on Bone Health, Vascular Calcification, and Health-Related Quality of Life in Hemodialysis Patients with Osteoporosis: A Prospective Observational Study. J. Clin. Med..

[88] Simonini M., Bologna A., Vezzoli G. (2024). Is denosumab an efficient and safe drug for osteoporosis in dialysis patients? Considerations and state of the art about its use in this setting. Int. Urol. Nephrol..

[89] Festuccia F., Jafari M.T., Moioli A., Fofi C., Barberi S., Amendola S., Sciacchiano S., Punzo G., Menè P. (2017). Safety and efficacy of denosumab in osteoporotic hemodialysed patients. J. Nephrol..

[90] Takeuchi Y., Tanaka S., Kuroda T., Hagino H., Mori S., Soen S. (2024). Association between renal function and fracture incidence during treatment with teriparatide or alendronate: An exploratory subgroup analysis of the Japanese Osteoporosis Intervention Trial-05. Osteoporos. Int..

[91] Nishikawa A., Yoshiki F., Taketsuna M., Kajimoto K., Enomoto H. (2016). Safety and effectiveness of daily teriparatide for osteoporosis in patients with severe stages of chronic kidney disease: Post hoc analysis of a postmarketing observational study. Clin. Interv. Aging.

[92] Miller P.D., Hattersley G., Riis B.J., Williams G.C., Lau E., Russo L.A., Alexandersen P., Zerbini C.A.F., Hu M.Y., Harris A.G. (2017). Effect of Abaloparatide vs. Placebo on New Vertebral Fractures in Postmenopausal Women with Osteoporosis: A Randomized Clinical Trial. JAMA.

[93] Battaglia Y., Baciga F., Bulighin F., Amicone M., Mosconi G., Storari A., Brugnano R., Pozzato M., Motta D., D’alessandro C. (2024). Physical activity and exercise in chronic kidney disease: Consensus statements from the Physical Exercise Working Group of the Italian Society of Nephrology. J. Nephrol..

[94] Cardoso D.F., Marques E.A., Leal D., Ferreira  A., Baker L.A., Smith A.C., Viana J.L. (2020). Impact of physical activity and exercise on bone health in patients with chronic kidney disease: A systematic review of observational and experimental studies. BMC Nephrol..

[95] Wu M., Li H., Sun X., Zhong R., Cai L., Chen R., Madeniyet M., Ren K., Peng Z., Yang Y. (2025). Aerobic exercise prevents renal osteodystrophy via irisin-activated osteoblasts. JCI Insight.

[96] Valenzuela P.L., Castillo-García A., Saco-Ledo G., Santos-Lozano A., Lucia A. (2024). Physical exercise: A polypill against chronic kidney disease. Nephrol. Dial. Transplant..

[97] Heiwe S., Jacobson S.H. (2011). Exercise training for adults with chronic kidney disease. Cochrane Database Syst. Rev..

[98] Magalhães de Castro B., Dos Santos Rosa T., de Araújo T.B., de Luca Corrêa H., de Deus L.A., Neves R.V.P., Reis A.L., Dos Santos R.L., da Silva Barbosa J.M., de Sousa Honorato F. (2024). Effects of cluster set resistance training on bone mineral density and markers of bone metabolism in older hemodialysis subjects: A pilot study. Bone.

[99] Marinho S.M., Moraes C., Barbosa J.E., Carraro Eduardo J.C., Fouque D., Pelletier S., Mafra D. (2016). Exercise Training Alters the Bone Mineral Density of Hemodialysis Patients. J. Strength Cond. Res..

[100] Petrauskiene V., Hellberg M., Svensson P., Zhou Y., Clyne N. (2023). Bone mineral density after exercise training in patients with chronic kidney disease stages 3 to 5: A sub-study of RENEXC—A randomized controlled trial. Clin Kidney J..

[101] Castillo R.F., Pérez R.G., González A.L. (2024). Beneficial effects of physical exercise on the osteo-renal Klotho-FGF-23 axis in Chronic Kidney Disease: A systematic review with meta-analysis. Int. J. Med. Sci..

[102] Bishop N.C., Burton J.O., Graham-Brown M.P.M., Stensel D.J., Viana J.L., Watson E.L. (2023). Exercise and chronic kidney disease: Potential mechanisms underlying the physiological benefits. Nat. Rev. Nephrol..

[103] Shah A., Hashmi M.F., Aeddula N.R. (2025). Chronic Kidney Disease-Mineral Bone Disorder (CKD-MBD). 3 April 2024. StatPearls [Internet].

